# The World’s Workers

**Published:** 1859-12

**Authors:** 


### THE WORLD'S WORKERS

On a mellow afternoon of the early fall, we read a tract for the first time which put us on a train of thought having in it the elements of the beautiful and the sad. Beautiful, because it shows that there are grand workers on the earth's surface; and not the less grand because they "choose not to let the left hand know what the right hand doeth," working in secret and rejoicing in that work, not for the human applause it will bring, but for the good it may do humanity, and for the sufferings it may avert from the brother man, some unknown brother battling with life's toils and trials, its hardships and its bitter tears. Here is a man who is a stranger to the ineffable sweets of wife-love, but his heart must be in a good place, for it must love something, and it spreads itself out to cover all human kind.

This tract is published and scattered abroad in thousands at the expense of the composer, thus making himself one of the army, whose work is not to murder, but to save and happify, and bless. We re-publish the tract in the sad remembrance of that larger multitude of workers who are busy in pulling down, in destroying, in sowing the seeds of drunkenness, and prostitution, and crime:—

